# The Effect of Dexmedetomidine on Mortality in Patients with Acute Pancreatitis: A Retrospective Propensity Score Matching Analysis

**DOI:** 10.5152/tjg.2025.25331

**Published:** 2025-11-11

**Authors:** Hui Zhang, Hui-juan Wang, Wen-jing Tang, Yun-long Wu

**Affiliations:** Department of Critical Care Medicine, Lin-ping Campus of the Second Affiliated Hospital, Zhejiang University School of Medicine, Hangzhou, China

**Keywords:** Acute pancreatitis, dexmedetomidine, MIMIC-IV database, mortality rate

## Abstract

**Background/Aims:**

Acute pancreatitis (AP) is a prevalent gastrointestinal disorder, with its frequency rising annually, and the fatality rate in severe cases reaching 38%. Dexmedetomidine (DEX), possessing analgesic, sedative, anti-inflammatory, and anti-sympathetic properties, appears to be a viable pharmacological option for AP; however, the clinical correlation remains ambiguous. This study aimed to elucidate the potential of DEX in enhancing the prognosis of patients with AP.

**Materials and Methods:**

The Medical Information Mart for Intensive Care–IV database served as the foundation for this retrospective propensity score-matched cohort analysis. Participants with AP diagnoses were split into 2 groups according to whether they received DEX for the study. Propensity score matching (PSM) was used to align the baseline data for the 2 groups. A multivariate Cox proportional hazards regression model and Kaplan–Meier survival curves were used to assess the relationship between DEX treatment and the 60-day death rate in patients with AP. Subgroup analyses were undertaken to ensure the reliability of the findings.

**Results:**

There were 362 patients in this test period, 181 of whom were in the DEX group and 181 of whom were in the No-DEX group. The standardized mean differences of all baseline features were less than 0.1, and the *P*-value was greater than .05 after the PSM of the baseline data of the 2 patient groups. This suggests that the 2 patient groups were well-balanced following matching. The 2 groups’ 60-day mortality rates differed significantly, according to the Kaplan–Meier survival curve. The DEX group’s survival rate was higher than the No-DEX group’s at the same time point (hazard ratio [HR] (95% CI) = 0.048 (0.406-1.002), *P* = .048). To evaluate the effect of DEX on mortality in AP patients, multiple Cox regression models that adjusted for different factors were used. According to the fully adjusted model, DEX improves the prognosis of patients with AP. The HR for 60-day death in the matched group was 0.51 (95% CI: 0.30-0.88), *P* = .015.

**Conclusion:**

The current study revealed that DEX administration can decrease the 60-day death rate in individuals with AP. Nonetheless, the verification of this claim necessitates multicenter randomized controlled trials.

**Main Points**

- From a pathophysiological perspective, dexmedetomidine is a potential treatment for acute pancreatitis (AP) because of its sedative, analgesic, anti-inflammatory, and anti-sympathetic qualities.
- Dexmedetomidine can reduce the 60-day mortality rate in patients with AP.
- The association between dexmedetomidine administration and 60-day mortality in AP patients remains unaffected by subgroup variables, including gender, age, body mass index, and comorbidities.

## Introduction

Acute pancreatitis (AP) is an acute digestive disorder that disrupts homeostasis in pancreatic acinar tissues due to various factors, leading to premature activation of pancreatic enzymes and subsequent self-digestion of pancreatic tissue.^1^ A systematic review and meta-analysis indicated a global increase in AP incidence and hospitalization rates. The study reported that from 1961 to 2016, the incidence rose from 2.30% to 3.84% worldwide, continuing to escalate at an annual rate of 3.07%.^2^ The growing number of pancreatitis cases has placed a substantial and increasing burden on healthcare utilization and expenditures. Survey results indicated that the annual number of hospital admissions for AP in the United States was 255 130, with a median cost of $7941, culminating in a total expenditure of $3 019 327 516.^3^ The ongoing advancement of imaging technologies and the evolution of therapy paradigms have consistently improved AP diagnostic and therapeutic standards. The mortality rate for AP remains significantly elevated. Research findings reveal that the overall in-hospital death rate for AP is 3.5%, with fatality rates of 0.3%, 2.3%, and 38% for mild, moderate, and severe cases, respectively.^4^ Consequently, optimizing the management of AP and improving patient outcomes is an imperative aim.

Acute pancreatitis is a complex inflammatory disease that initially manifests as a sterile inflammatory response. Acinar and ductal cells may experience damage or necrosis due to multiple factors, resulting in the release of pancreatic enzymes into the surrounding tissue and subsequent local pancreatic injury.^5^ As the disease progresses, severe cases may develop systemic inflammatory response syndrome, extrapancreatic organ failure, or even death.^6^ Additionally, the pancreas is a gland with extensive nerve innervation, comprising sympathetic and parasympathetic efferent nerves, spinal and vagus nerve afferents, and innervation from the enteric nervous system.^7^ Pain stimuli and inflammatory responses can induce sympathetic nerve excitation, resulting in vascular endothelium damage, exacerbating pancreatic microcirculatory disorders, and worsening the condition. Abdominal pain is the predominant symptom observed in patients diagnosed with AP. A survey indicated that 97.3% of patients reported pain upon admission, with the intensity and duration of pain closely correlated to the severity of AP.^8^ The physiological mechanisms underlying pain in AP are highly complex, with different mechanisms leading to distinct types of pain: neuropathic pain, inflammatory pain, and nociceptive pain.^9^ The complexity of pain management in AP arises from the diverse mechanisms of pain associated with the condition. Clinical guidelines lack consistent recommendations for selecting various analgesic methods or medications.^10^

Dexmedetomidine (DEX) is a selective and potent α2-adrenoceptor agonist exhibiting superior analgesic properties. Research findings indicate that, compared to remifentanil, DEX offers superior analgesic effects, diminishes the incidence of severe postoperative pain, lowers morphine requirements, and extends the duration until the initial rescue analgesia is administered.^11^ Current recommendations for managing AP include stepwise and multimodal analgesia regimens. The incorporation of DEX in these regimens not only provides effective pain relief but also alleviates the adverse effects associated with other medications.^12^^,^^13^ Furthermore, DEX inhibits sympathetic nervous system excitation. Research shows that during DEX administration, norepinephrine levels decreased by 72% and epinephrine levels by 10%, which reduces early postoperative sympathetic nervous system activity.^14^ Additionally, DEX exhibits anti-inflammatory properties by decreasing the release of inflammatory factors such as tumor necrosis factor-α (TNF-α) and interleukin-6 (IL-6) and promoting acetylcholine release, thereby offering biological protection for organs.^15^

The majority of severe acute pancreatitis (SAP) patients admitted to the intensive care unit (ICU) experience pain, delirium, and sympathetic nervous system hyperactivity. Dexmedetomidine appears to be a suitable pharmacological agent for managing AP. However, there is a lack of clinical studies regarding the use of DEX in AP patients, and the evidence is primarily drawn from animal research. Therefore, clinical data were extracted and analyzed from the Medical Information Mart for Intensive Care (MIMIC)-IV registry of AP patients who received at least 1 dose of DEX to evaluate whether its administration can improve the prognosis of these patients.

## Materials and Methods

### Data Source

The Medical Information Mart for Intensive Care–IV (MIMIC-IV) database serves as the basis for this retrospective propensity score-matched cohort analysis. The Beth Israel Deaconess Medical Center’s computerized medical records are the source of the publicly accessible MIMIC-IV database. This collection of modern electronic health records spans 10 years, from 2008 to 2019. MIMIC-IV v3.1 includes 364 627 unique individuals, representing 94 458 unique ICU stays and 546 028 hospitalizations. After reviewing the Beth Israel Deaconess Medical Center’s patient data collection and research resource development, the Institutional Review Board approved the data-sharing effort and waived informed consent.^16^ The database is accessible to those who pass the Collaborative Institutional Training Initiative exam (author Hui Zhang’s certification number is 64028164). The Strengthening the Reporting of Observational Studies in Epidemiology statement was followed in the preparation of the manuscript.^17^ This study was approved by the Ethics Committee of the Lin-ping Campus of the Second Affiliated Hospital, Zhejiang University School of Medicine (approval no.: 2024016; date: March 18, 2024).

### Study Population

The study encompassed all patients admitted to the ICU diagnosed with AP, as defined by the diagnostic criteria of the International Statistical Classification of Diseases, 9th and 10th Revisions (ICD-9 and ICD-10). To ensure consistency, information was only extracted on patients admitted to the ICU for the first time during their hospitalization. Patients discharged or deceased within 48 hours of ICU admission and those under 18 will be excluded from the study. Depending on whether or not they had received DEX treatment during hospitalization, the enrolled patients were divided into the DEX and no-DEX groups.

### Data Extraction and Outcomes

We used Navicat Premium (version 17.0.8) (PremiumSoft CyberTech Limited; Hong Kong, China) and Structured Query Language to extract and process data from the MIMIC-IV database. The following traits were taken out: (1) demographic data, such as age, gender, and body mass index (BMI); (2) comorbidities, such as diabetes, hypertension, liver disease, sepsis, chronic lung illness, and renal disease; (3) vital signs at admission, such as heart rate, respiratory rate, temperature, mean blood pressure (MBP), and peripheral oxygen saturation (SpO_2_); (4) clinical scores, such as the Charlson comorbidity index (CCI), acute physiology score III, logistic organ dysfunction system (LODS), Oxford acute severity of illness score (OASIS), and sequential organ failure assessment (SOFA); (5) laboratory parameters, including white blood cell (WBC), platelets, hemoglobin, potential hydrogen (pH), anion gap, bicarbonate, alanine aminotransferase (ALT), aspartate aminotransferase (AST), total bilirubin, albumin, blood urea nitrogen (BUN), creatinine, glucose, calcium, sodium, potassium, international normalized ratio (INR), prothrombin time (PT), and partial thromboplastin time (PTT). The main outcome measure was all-cause mortality at 60 days.

### Statistical Analysis

The mean ± SD was used to represent the measurement data that had a normal distribution. To compare the 2 groups, an independent samples t-test was employed. On the other hand, measurement data that did not fit a normal distribution were denoted by M (P25, P75), and group comparison was done using a non-parametric test called the Mann–Whitney *U*-test. The chi-square test or Fisher’s exact test was used to compare groups, and count data were displayed as the number of instances and percentages (%). Multiple imputation by chained equations was used to ensure unbiased estimates in cases where the data contained missing values of less than 20%. Variables with more than 20% missing data were not included in the study.

In order to reduce confounding bias, the statistical technique of propensity score matching (PSM) was applied to the balanced baseline data between the 2 patient groups. In order to determine the propensity score for each patient, a logistic regression model was utilized. The closest neighbor approach was used to generate the matching based on a 1:1 ratio with a caliper width of 0.05 without replacement. Following PSM, *P*-values and standardized mean differences (SMDs) were used to assess how well the 2 groups’ characteristics were balanced. When the SMD of a variable is less than 0.1, it is deemed to be balanced between the groups.

Kaplan–Meier survival curves were plotted to track changes in survival rates over time for the DEX and no-DEX groups. The log-rank test was then applied to see if there were any variations between the 2 groups’ survival curves.

Cox regression analysis was utilized to establish multiple models, controlling for various confounding factors, to verify whether DEX use can reduce the 60-day mortality rate in patients. Model 1 (unadjusted): Only the dependent variable, DEX, and the outcome variable, 60-day mortality, were included in the analysis model, with no covariates. Model 2: In addition to Model 1, the following variables were included: age, gender, BMI, and comorbidities. Model 3: In addition to Model 2, vital signs at admission were incorporated. Model 4: Along with Model 3, patient clinical scores were factored in. Model 5 (fully adjusted): Besides Model 4, laboratory parameter results were included.

Furthermore, the objective was to investigate the correlation between DEX utilization and survival across diverse population groups. To this end, a series of subgroup analyses was conducted, incorporating variables such as age, sex, BMI, the presence of chronic pulmonary disease, liver disease, diabetes, hypertension, renal disease, and sepsis.

All statistical analyses in this study were conducted using R software (version 4.4.3) (University of Auckland; Auckland, New Zealand). Statistical significance was deemed achieved when the *P*-value was less than .05.

## Results

### Population Selection

**Figure 1.**
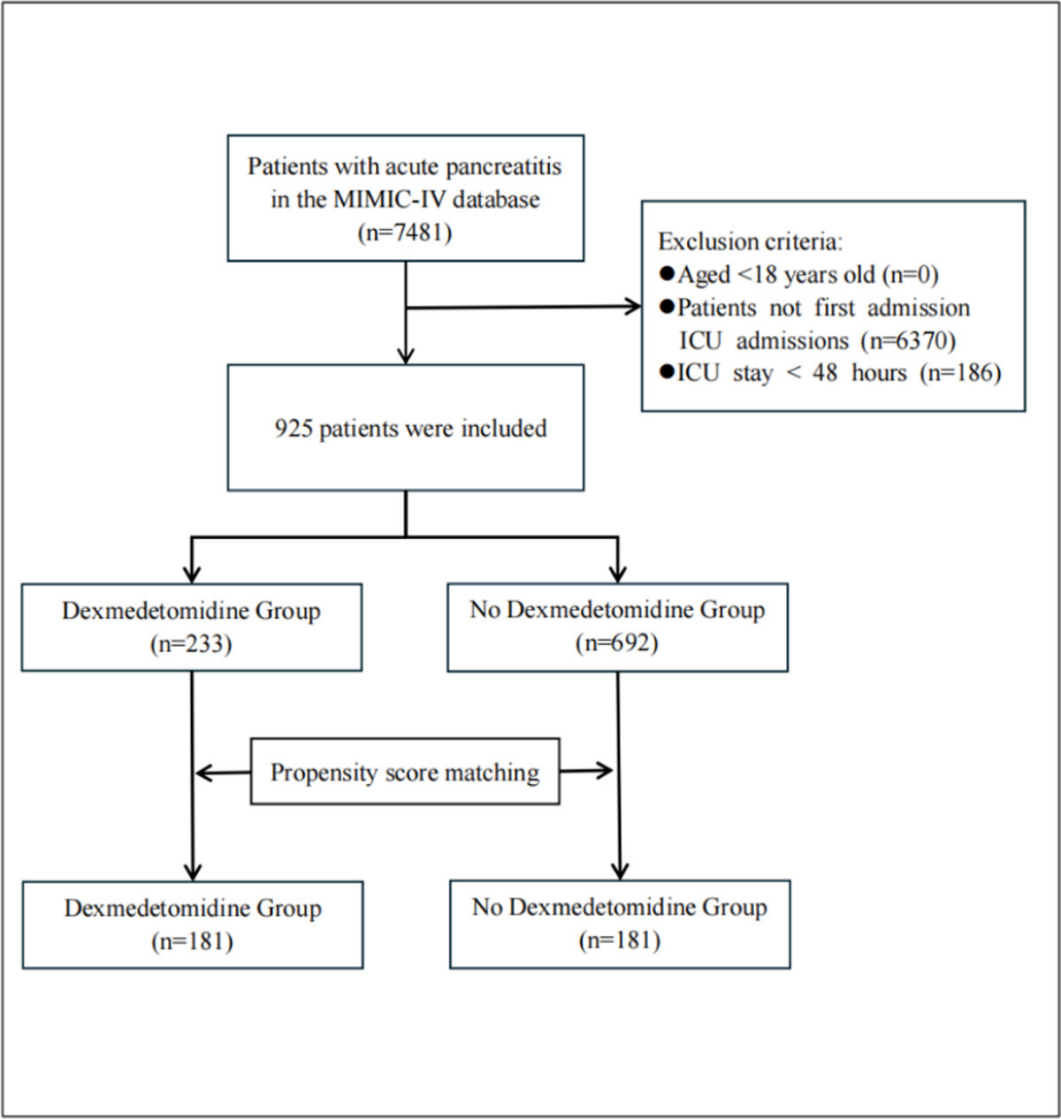
Inclusion and exclusion flowchart of the study.

The MIMIC-IV database includes information on 7481 patients diagnosed with AP. After excluding 6370 cases of non-first-time ICU admissions and 186 patients in the ICU for less than 48 hours, 925 patients were selected for this study. Patients were divided into 2 groups based on their prior experience with DEX: the DEX group (n = 233) and the no-DEX group (n = 692). Following PSM, the sample consisted of 181 patients across the 2 groups (Figure 1).

### Baseline Characteristics

Patients with AP in the DEX group had higher BMI, heart rate, respiratory rate, temperature, SOFA, APACHE II, LODS, OASIS, creatinine, and PTT than those in the no-DEX group. The no-DEX group showed a higher proportion of males, a greater prevalence of liver disease and sepsis, and higher age, CCI, pH, bicarbonate, ALT, albumin, and calcium levels. No statistically significant differences were found between the 2 groups regarding chronic pulmonary disease, diabetes, hypertension, renal disease, MBP, SpO2, WBC, platelets, hemoglobin, anion gap, AST, total bilirubin, BUN, glucose, sodium, potassium, INR, and PT. After performing PSM on the baseline data of the 2 patient groups, the SMD of all baseline characteristics was less than 0.1, and the *P-*value was greater than .05, indicating that the 2 patient groups were well-balanced after matching (Table 1).

### Survival Analysis

**Figure 2.**
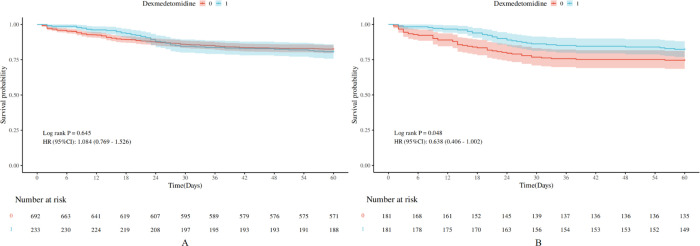
The KM survival curve illustrates the variations in the 60-day survival status of 2 cohorts of acute pancreatitis patients before (A) and after (B) using propensity score matching.

Figure 2 illustrates a survival curve analysis investigating the correlation between DEX administration and 60-day survival rates in patients with AP. The Kaplan-Meier survival analysis indicated that the survival curve for the DEX cohort consistently exceeded that of the no-DEX cohort over time. Furthermore, the log-rank test produced a *P*-value of .048, signifying a statistically significant difference in survival between the 2 cohorts. Consequently, DEX usage in patients with AP significantly improves survival outcomes (Figure 2).

### Cox Proportional Hazards Regression Model

Single-factor and multi-factor Cox proportional hazards regression models were utilized to create various models by adjusting for different covariates. The analysis revealed that in the unadjusted model of the single-factor Cox regression, DEX usage did not influence the 60-day mortality rate in AP patients (HR = 0.64, 95% CI (0.41-1.00), *P* = .051). However, after adjusting for potential confounding factors affecting prognosis, the fully adjusted model that included demographic characteristics, comorbidities, vital signs at admission, clinical scores, and laboratory parameters (Model 5) was established. The results indicated HR = 0.51, 95% CI (0.30-0.88), *P* = .015, suggesting that DEX usage positively impacts survival outcomes in AP patients, reinforcing the reliability of the analysis results (Table 2).

### Subgroup Analyses

**Figure 3.**
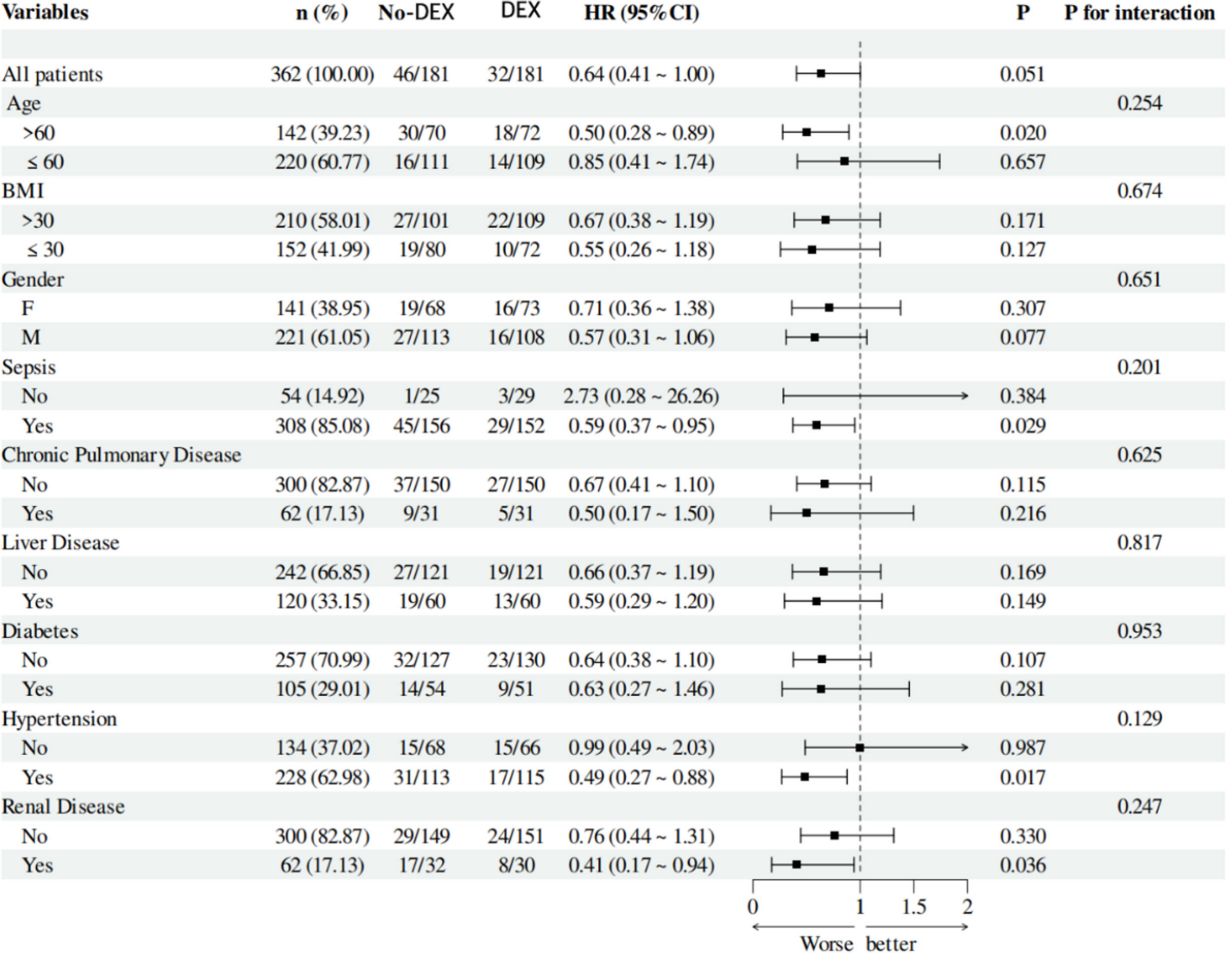
Subgroup examination of the correlation between dexmedetomidine administration and outcomes in several patient subgroups with acute pancreatitis.

Patients were categorized into subgroups based on age, gender, BMI, and comorbidities. Subgroup analysis indicated that patients over 60 with sepsis, hypertension, and kidney disease exhibited hazard ratios (HRs) of 0.50, 0.59, 0.49, and 0.41, all with *P*-values below .05. This implies that these patients experienced worse outcomes. However, a comprehensive analysis of all subgroup factors and their interactions with DEX usage revealed that the *P*-values for interaction exceeded .05, signifying no interaction effects among subgroups. This indicates that DEX efficacy is not affected by the aforementioned subgroup factors concerning 60-day survival rates, affirming the robustness of the analytical findings (Figure 3).

## Discussion

This study utilized data from the MIMIC-IV database to investigate the correlation between DEX and the prognosis of individuals with AP. Propensity score matching was employed to balance the baseline characteristics of the 2 cohorts, generate Kaplan–Meier survival curves, and develop multiple Cox proportional hazards regression models. Ultimately, it was determined that DEX improved the 60-day survival rate of individuals with AP. Despite variations in prognosis among subgroups of patients with AP, it was found that the interaction *P*-values for each subgroup exceeded .05. This indicates that the efficacy of DEX remains consistent across different subgroups of patients with AP.

The etiology of AP is varied, and its pathogenesis remains incompletely understood. Along with early trypsinogen activation, defective calcium signaling, poor autophagy, endoplasmic reticulum stress, the unfolded protein response, and mitochondrial dysfunction are key contributors to the pathophysiology of AP.^18^ The variety of causes and the complexity of processes have led to differing views on the pathophysiological process of AP. It is well acknowledged that the early stage of AP entails a sterile inflammatory response.^19^ Due to multiple factors, excessive accumulation of calcium ions in pancreatic cells reduces adenosine triphosphate synthesis in mitochondria, leading to injury or necrosis of acinar and ductal cells.^5^ During the initial phases of the disease, the cells in the affected pancreas produce damage-associated molecular patterns, which act as intrinsic danger signals, prompting the body to activate an immunological response. This results in the release of significant quantities of pro-inflammatory mediators and the infiltration of immune cells, worsening the inflammatory cascade and inducing uncontrolled or dysregulated immune responses, ultimately leading to systemic inflammatory response syndrome or even multiple organ dysfunction syndrome.^6^^,^^20^ The α2-adrenergic receptor is a G protein-coupled receptor composed of 3 distinct subtypes (α2A, α2B, and α2C) located throughout the central and peripheral nervous systems, as well as in various organs and tissues.^21^ Dexmedetomidine is a highly selective α2-adrenergic receptor agonist that not only possesses analgesic, sedative-hypnotic, and anti-sympathetic effects but also demonstrates anti-inflammatory and anti-apoptotic properties, offering protective benefits to organs such as the nervous system, lungs, kidneys, liver, and intestines.^15^ Numerous high-quality clinical trials have shown that DEX can positively influence conditions such as sepsis, acute respiratory distress syndrome (ARDS), and acute kidney injury (AKI). An analysis demonstrated that compared with benzodiazepines, DEX reduces mortality in patients with sepsis. Although there was no significant difference in mortality rates compared with propofol, DEX significantly reduced the inflammatory response in patients with sepsis.^22^ In another propensity score-matched cohort study, patients with ARDS who received DEX had a lower risk of death compared to those who received midazolam and propofol.^23^ Additionally, a meta-analysis indicated that while DEX increases the incidence of bradycardia, it reduces the risk of postoperative AKI and postoperative delirium and shortens ICU and hospital stays.^24^

Considering the pathogenesis of pancreatitis and the physiological effects of DEX, it appears that DEX is a suitable pharmacological option for managing AP. Most research regarding DEX and AP remains in the animal experimentation phase. A study involving animals demonstrated that DEX reduces SAP-induced pancreatic injury, neutrophil and macrophage infiltration, and oxidative stress. Transcriptomics and molecular biology were employed to clarify the impact of DEX on necrotic apoptosis in SAP and the molecular pathways involved in these effects.^25^ A separate animal study indicated that DEX can reduce systemic inflammatory responses and local pancreatic injury caused by SAP in rats via a cholinergic anti-inflammatory pathway mediated by vagal and alpha-7 nicotinic acetylcholine receptor dependent mechanisms.^26^ Most studies in clinical settings have concentrated on the application of DEX in pancreatic surgery. A single-center randomized controlled trial demonstrated that administering DEX to elderly patients undergoing major pancreatic surgery could reduce inflammatory responses and lower the incidence of AKI and pulmonary complications.^27^ A separate study examined the application of DEX in patients undergoing laparoscopic pancreaticoduodenectomy (LPD). The findings indicated DEX administration during surgery may mitigate early inflammatory responses after LPD.^28^ This study analyzed data from the MIMIC-IV database and concluded that DEX may enhance the 60-day mortality rate in patients with AP. Propensity score matching was employed to control for confounding factors and improve comparability between the 2 groups. Following PSM, the baseline characteristics of the 2 groups were equilibrated. The Kaplan-Meier survival curves indicated that patients administered DEX exhibited improved survival outcomes. Several regression models were developed to confirm this finding while controlling for various covariates. In the fully adjusted model, the HR for 60-day mortality in the matched population was 0.51 (95% CI: 0.30-0.88), *P* = .015, indicating that DEX improves the prognosis of patients with AP.

Multiple studies indicate that sepsis significantly impacts the prognosis of patients with AP. Acute pancreatitis initially presents as a sterile inflammation; however, approximately 40% of patients develop an infection in the later stages of the condition.^29^ Additionally, studies reveal that the mortality rate for AP patients complicated by organ failure and sepsis is 35.2%, whereas the mortality rate for sterile necrosis and organ failure is 19.8%. The mortality rate for patients with sepsis who do not exhibit organ failure is 1.4%. Organ failure and sepsis-related necrosis increase the mortality rate associated with necrotizing pancreatitis.^30^ Acute kidney injury frequently occurs in cases of AP and is linked to the prognosis of patients with this condition. Studies show that among hospitalized patients with AP, the AKI group has a significantly higher mortality rate than the no-AKI group (rates of 8.8% and 0.7%, respectively; *P* < .01).^31^ To evaluate the possible impact of these factors on the findings of this study, a subgroup analysis was carried out. According to the subgroup analysis, the administration of DEX had a significant impact on the 60-day survival rate among patients who were over 60 (HR = 0.50, 95% CI (0.28-0.89), *P* = .020), had concomitant sepsis (HR = 0.59, 95% CI (0.37-0.95), *P* = .029), had hypertension (HR = 0.49, 95% CI (0.27-0.88), *P* = .017), or had renal insufficiency (HR = 0.41, 95% CI (0.17-0.94), *P* = .036). The* P-*value for interaction was more than .05 when grouping characteristics such as patient age, BMI, gender, presence of sepsis, chronic lung disease, liver disease, diabetes, hypertension, and renal disease were taken into account. This suggests that there is no substantial correlation between the 60-day mortality rate and the use of DEX and these confounding factors. As a result, these factors did not affect the association between DEX use and the 60-day survival rate, confirming the validity of the analytical findings.

This study has certain limitations. Firstly, it is a clinical retrospective analysis. Despite employing PSM to balance the baseline characteristics of the 2 groups, residual bias remains. Further validation through randomized controlled trials is necessary. Secondly, analysis of the MIMIC-IV database revealed that the missing C-reactive protein (CRP) rate among the enrolled patients exceeded 50%. Imputing missing values would significantly alter the data distribution; therefore, CRP was omitted as a parameter. Moreover, the MIMIC database’s lack of commonly used clinical inflammatory markers, including TNF-α, IL-6, and procalcitonin, limited the ability to gather and analyze these data, impacting the comprehensiveness of the experimental analysis. Additionally, as the data for this study were obtained from a single-center critical care database, its characteristics, such as geographical location, ethnicity, economic conditions, and treatment options, do not represent those of the critical care patient population in different regions. Although this study observed that dexmedetomidine is widely used in patients with AP at Beth Israel Deaconess Medical Center, since dexmedetomidine is not the first-line analgesic or therapeutic drug in the treatment regimen for AP, it may not be widely adopted in different regions, which could affect the generalizability and objectivity of the study results. Therefore, prospective, global, multicenter cohort studies should be conducted in the future to validate whether the use of dexmedetomidine can influence the prognosis of AP patients.

The current study revealed that dexmedetomidine administration can decrease the 60-day death rate in individuals with AP. Nonetheless, the verification of this claim necessitates multicenter randomized controlled trials.

## Figures and Tables

**Table 1.** Baseline Features of the Original and Propensity Score Matching Populations

Variable	Before PSM					After PSM				
	Total (n = 925)	No-DEX (n = 692)	DEX (n = 233)	*P*	SMD	Total (n = 362)	No-DEX (n = 181)	DEX (n = 181)	*P*	SMD
Gender, male, n (%)	532 (57.51)	383 (55.35)	149 (63.95)	.022	0.179	221 (61.05)	113 (62.43)	108 (59.67)	.590	−0.056
Age (years)	58.57 (45.65, 72.17)	61.11 (47.32, 74.53)	52.20 (40.21, 65.63)	<.001	−0.454	55.24 (43.35~67.74)	55.64 (44.32~67.48)	54.56 (43.02~67.77)	.875	−0.013
BMI (kg/m^2^)	29.27 (25.65, 33.95)	28.46 (25.12, 33.38)	31.28 (28.09, 36.17)	<.001	0.406	30.85 (26.79~36.05)	30.81 (25.76~37.17)	30.86 (27.33~35.04)	.564	0.019
Comorbidities, n (%)										
Chronic pulmonary disease	178 (19.24)	140 (20.23)	38 (16.31)	.189	−0.106	62 (17.13)	31 (17.13)	31 (17.13)	1.000	0.000
Liver disease	285 (30.81)	199 (28.76)	86 (36.91)	.020	0.169	120 (33.15)	60 (33.15)	60 (33.15)	1.000	0.000
Diabetes	278 (30.05)	218 (31.50)	60 (25.75)	.098	−0.132	105 (29.01)	54 (29.83)	51 (28.18)	.728	−0.037
Hypertension	576 (62.27)	438 (63.29)	138 (59.23)	.268	−0.083	228 (62.98)	113 (62.43)	115 (63.54)	.828	0.023
Renal disease	155 (16.76)	118 (17.05)	37 (15.88)	.679	−0.032	62 (17.13)	32 (17.68)	30 (16.57)	.78	−0.030
Sepsis	636 (68.76)	432 (62.43)	204 (87.55)	<.001	0.761	308 (85.08)	156 (86.19)	152 (83.98)	.555	−0.060
Vital signs at admission										
Heart rate (bpm)	95.60 (82.69, 108.62)	94.38 (81.09, 107.71)	99.25 (85.92, 112.40)	.008	0.198	98.17 ± 19.15	97.62 ± 19.39	98.71 ± 18.94	.590	0.057
MBP (mmHg)	79.70 (72.34, 90.92)	80.10 (72.49, 91.63)	78.64 (71.70, 86.78)	.083	−0.163	78.67 (71.51~88.63)	77.88 (72.12~88.12)	79.27 (71.16~88.77)	.994	−0.019
Resp rate (/min)	20.70 (17.96, 24.13)	20.45 (17.75, 23.71)	21.92 (18.56, 24.85)	<.001	0.251	21.71 (18.57~24.86)	21.29 (18.61~24.84)	21.92 (18.58~24.96)	.638	0.025
Temperature (°C)	36.94 (36.66, 37.36)	36.90 (36.63, 37.25)	37.15 (36.78, 37.62)	<.001	0.360	37.06 (36.71~37.51)	36.99 (36.67~37.57)	37.13 (36.75~37.49)	.425	0.057
SpO_2_ (%)	96.21 (94.83, 97.67)	96.17 (94.81, 97.64)	96.33 (94.88, 97.77)	.658	0.006	96.32 (94.81~98.04)	96.39 (94.75~98.24)	96.27 (94.86~97.84)	.570	−0.012
Clinical scores										
SOFA	6.00 (3.00, 9.00)	5.00 (2.00, 8.00)	8.00 (5.00, 11.00)	<.001	0.559	7.00 (4.00~11.00)	7.00 (4.00~11.00)	7.00 (4.00~11.00)	.973	−0.023
APSIII	50.00 (37.00, 70.00)	47.00 (35.00, 65.00)	58.00 (47.00, 79.00)	<.001	0.419	57.00 (43.00~78.00)	55.00 (41.00~78.00)	57.00 (45.00~78.00)	.53	−0.009
LODS	5.00 (2.00, 8.00)	4.00 (2.00, 7.00)	7.00 (4.00, 9.00)	<.001	0.704	6.00 (4.00~9.00)	6.00 (4.00~9.00)	6.00 (4.00~9.00)	.795	−0.077
OASIS	33.00 (27.00, 40.00)	31.50 (26.00, 38.00)	37.00 (31.00, 43.00)	<.001	0.616	37.470 ± 8.879	37.76 ± 9.502	37.18 ± 8.225	.535	−0.071
CCI	3.00 (2.00, 6.00)	4.00 (2.00, 6.00)	3.00 (1.00, 5.00)	.021	−0.186	3.00 (1.00~5.00)	3.00 (1.00~5.00)	3.00 (2.00~5.00)	.590	0.056
Laboratory parameters										
WBC (×10^9^)	11.78 (8.50, 16.43)	11.80 (8.52, 16.06)	11.65 (8.33, 17.18)	.589	0.015	11.65 (8.34~17.16)	11.65 (8.33~17.13)	11.65 (8.65~17.17)	.779	0.033
Platelet (×10^9^)	198.75 (141.67, 273.35)	203.00 (144.38, 275.18)	191.75 (138.00, 266.00)	.227	−0.071	189.57 (135.57~266.13)	188.50 (133.86~259.80)	192.00 (138.00~273.29)	.878	−0.014
Hemoglobin (g/L)	11.35 (9.90, 12.90)	11.42 (10.04, 12.88)	11.16 (9.45, 13.03)	.166	−0.075	11.20 (9.73~12.81)	11.25 (9.90~12.84)	11.16 (9.63~12.70)	.490	−0.053
Ph	7.36 (7.30, 7.42)	7.36 (7.31, 7.42)	7.33 (7.28, 7.40)	.003	−0.182	7.35 (7.29~7.41)	7.35 (7.29~7.41)	7.34 (7.29~7.41)	.611	−0.033
Aniongap (mmol/L)	14.80 (12.50, 17.60)	15.00 (12.67, 17.60)	14.50 (12.00, 17.50)	.286	−0.035	14.79 (12.50~17.76)	15.33 (13.00~18.00)	14.33 (12.00~17.50)	.185	−0.054
Bicarbonate (mmol/L)	21.50 (18.33, 24.33)	21.67 (18.50, 24.50)	20.80 (17.75, 24.00)	.045	−0.131	20.85 ± 4.59	20.99 ± 4.36	20.71 ± 4.81	.565	−0.058
ALT (U/L)	49.67 (24.00, 136.33)	54.50 (23.88, 151.62)	42.50 (24.33, 99.00)	.043	−0.342	47.75 (24.50~135.33)	53.50 (24.00~165.00)	44.00 (25.00~118.00)	.234	−0.051
AST (U/L)	73.50 (35.00, 177.50)	71.00 (34.38, 179.38)	78.00 (37.50, 162.50)	.328	−0.122	78.00 (39.00~180.63)	79.00 (41.67~178.33)	78.00 (38.00~191.00)	.953	0.023
Bilirubin Total (μmol/L)	1.10 (0.60, 3.20)	1.12 (0.60, 3.12)	1.10 (0.60, 3.25)	.967	0.102	1.10 (0.60~2.90)	1.20 (0.60~2.70)	1.08 (0.55~2.90)	.355	0.041
Albumin (g/L)	3.00 (2.55, 3.50)	3.10 (2.60, 3.50)	2.90 (2.40, 3.40)	.002	−0.234	2.90 (2.44~3.41)	2.85 (2.40~3.35)	2.85 (2.40~3.35)	.333	0.101
BUN (mmol/L)	20.00 (12.00, 37.00)	19.67 (11.63, 36.35)	21.60 (13.00, 37.83)	.313	0.041	22.00 (12.92~38.75)	22.50 (12.33~39.86)	22.00 (13.00~38.20)	.798	−0.065
Creatinine (μmol/L)	1.05 (0.73, 1.95)	1.00 (0.70, 1.83)	1.28 (0.80, 2.25)	.009	0.118	1.13 (0.73~2.13)	1.05 (0.73~2.13)	1.27 (0.75~8.37)	.531	−0.034
Glucose (mmol/L)	134.95 (109.00, 170.50)	133.54 (107.07, 168.37)	137.50 (114.22, 173.50)	.062	−0.723	134.90 (111.43~176.81)	133.75 (109.80~176.83)	137.10 (114.67~175.20)	.403	0.054
Calcium (mmol/L)	7.99 (7.43, 8.50)	8.00 (7.50, 8.51)	7.86 (7.32, 8.37)	.023	−0.206	7.84 (7.35~8.37)	7.75 (7.30~8.37)	7.90 (7.38~8.37)	.49	−0.011
Sodium (mmol/L)	138.00 (135.00, 140.75)	138.00 (135.00, 140.75)	137.60 (134.33, 141.00)	.454	−0.053	138.00 (135.33~141.41)	138.50 (136.33~141.33)	137.67 (135.00~141.50)	.076	−0.126
Potassium (mmol/L)	4.05 (3.70, 4.47)	4.03 (3.71, 4.40)	4.10 (3.70, 4.67)	.100	0.122	4.05 (3.70~4.57)	4.00 (3.73~4.43)	4.08 (3.70~4.67)	.589	0.06
INR	1.30 (1.15, 1.60)	1.30 (1.15, 1.60)	1.30 (1.20, 1.60)	.278	−0.055	1.30 (1.19~1.60)	1.33 (1.20~1.60)	1.30 (1.20~1.60)	.852	0.056
PT (s)	14.45 (13.00, 17.00)	14.45 (12.99, 17.00)	14.40 (13.10, 17.20)	.813	−0.041	14.60 (13.10~17.55)	14.82 (13.23~17.50)	14.20 (13.05~17.53)	.265	0.021
PTT (s)	30.90 (27.10, 38.20)	30.70 (26.87, 37.00)	32.60 (28.00, 40.23)	.005	0.148	31.38 (27.40~40.66)	31.35 (27.15~40.60)	31.45 (27.53~40.23)	.715	0.032

ALT, alanine aminotransferase; APSIII, acute physiology score III; AST, aspartate aminotransferase; BMI, body mass index; BUN, blood urea nitrogen; CCI, charlson comorbidity index; DEX, dexmedetomidine; INR, international normalized ratio; LODS, logistic organ dysfunction system; MBP, mean blood pressure; OASIS, oxford acute severity of illness score; Ph, potential hydrogen; PSM, propensity score matching; PT, prothrombin time; PTT, partial thromboplastin time; SMD, standardized mean difference; SOFA, sequential organ failure assessment; WBC, white blood cell.

**Table 2.** Results of Cox Proportional Hazard Models

Variables	Model 1		Model 2		Model 3		Model 4		Model 5	
	HR (95% CI)	*P*	HR (95% CI)	*P*	HR (95% CI)	*P*	HR (95% CI)	*P*	HR (95% CI)	*P*
Dexmedetomidine	0.64 (0.41 ~ 1.00)	.051	0.50 (0.31 ~ 0.82)	.006	0.50 (0.31 ~ 0.82)	.006	0.50 (0.31 ~ 0.82)	.006	0.51 (0.30 ~ 0.88)	.015

HR, hazard ratio.

## Data Availability

The data that support the findings of this study are available on request from the corresponding author.
